# Readability of Online Information for Parents Concerning Paediatric In-Toeing: An Analysis of the Most Popular Online Public Sources

**DOI:** 10.7759/cureus.57268

**Published:** 2024-03-30

**Authors:** Sahil S Shet, Ben Murphy, Sinead Boran, Colm Taylor

**Affiliations:** 1 Orthopaedics, Cork University Hospital, Cork, IRL

**Keywords:** pigeon toeing, readability, femoral anteversion, metatarsus adductus, in-toeing

## Abstract

Background

Parents often access online resources to educate themselves on their child’s condition. In-toeing, also referred to as pigeon toeing, is a common paediatric condition that has a variety of causes and is often a cause of concern for parents. With the increasing usage of the internet, parents of children with this condition may look to the web for answers. However, to be understood by the average adult, online health information must be written at an elementary school reading level. We hypothesised that currently available online resources regarding in-toeing would score poorly on objective measures of readability and understandability.

Methods

Patient education materials were identified via three commonly used online search engines (Google.com, Yahoo.com, and Bing.com). The terms “intoeing” and “pigeon toeing” were used for the search. From the top 50 search results, websites were included if directed at educating patients and their families regarding in-toeing. News articles, non-text material (video), industry websites, and articles not related to in-toeing were excluded. The readability was analysed using a specialised website www.readable.com to produce the following three scores: Gunning Fog Index (GFI), Flesch Reading Ease (FRE), and Flesch-Kincaid Grade (FKG). Understandability was calculated using the 19-point Patient Education Materials Assessment Tool (PEMAT).

Results

After removing duplicates, 84 unique websites were assessed for inclusion. A total of 48 websites and articles (57.14%) met the inclusion criteria. Of note, 23 articles out of 84 (27.38%) were excluded as they were intended for healthcare professionals. The means for the FRE, FKG, and GFI were 57.92 (±12.26), 7.92 (±1.91), and 9.35 (±2.36), respectively. Less than half of online resources had an FRE score at or higher than the recommended reading level for the general population. Mean understandability scores were 69.63% (±11.55%), with only 45.83% of articles being greater than the 70% requirement of adequate understandability.

Conclusion

Overall, online in-toeing educational materials scored poorly with respect to readability and understandability. Given the popularity of online resources in patient education, we should seek to improve this situation. Articles that are easier to read are thus more accessible to the general public and will aid in the shared decision-making process. Improved patient and parent satisfaction and overall standard of care can be expected.

## Introduction

In modern medicine, patients often utilise the internet to gain a better understanding of various health conditions. A recent study conducted at the University of Virginia found that over 60% of orthopaedic patients had used the internet for the purposes of obtaining orthopaedic information [1,2]. However, concerns have been raised in previous studies about the quality of material available to surgical patients online [3,4]. Health literacy is defined as “the degree to which individuals have the capacity to obtain, process, and understand basic health information and services needed to make appropriate health decisions” [5]. It is well established that poor health literacy can contribute to poor health, high risk of mortality, ineffective use of healthcare and health disparities [6]. Therefore, there has been a push towards increasing health literacy among patients by assessing and improving the healthcare material available online [7].

To improve the quality of material available online to patients, we must first examine the average literacy levels amongst the population in question. In Ireland, a 2012 Organisation for Economic Co-operation and Development (OECD) report found that 17.9% of adults are at or below level 1 on the literacy scale. The National Adult Literacy Agency (NALA) suggests that at this level, a person may be unable to understand basic written information. In the USA, adults have an average reading level equivalent to grade level 8 while over 50% of Canadians fall into the lowest two literacy categories. In the UK, just under one in six adults has the literacy of an 11-year-old [8]. Taking all this into consideration, the health materials available online should clearly be created with a reading level as low as possible while ensuring the quality of information is not affected. Although the recommended level of health-related materials for patients varies across studies, a 5th to 6th grade (USA) level has been suggested by several experts [9].

Multiple studies have explored the readability of materials pertaining to various orthopaedic topics; however, paediatric in-toeing has not been explored in this setting. Paediatric in-toeing is one of the most common reasons for referral to a paediatric orthopaedic surgeon. These referrals are primarily driven by parental concerns regarding the aesthetics of their child’s gait pattern. Therefore, multiple articles and resources should exist online to alleviate the concerns of these parents and improve health literacy. However, based on prior research, we hypothesised that the readability and understandability of online articles pertaining to paediatric in-toeing would be sub-standard.

## Materials and methods

Google, Yahoo, and Bing are the three most popular search engines used worldwide and thus were utilised for obtaining websites and articles for this study. Of the three resources, Google is by far the most popular with a market share of 83.49% in July 2023. Bing and Yahoo had market shares of 9.19% and 2.72%, respectively, for July 2023. The top 50 search results from each of the three search engines were analysed for inclusion. The search terms “intoeing” and “pigeon toeing” were used to obtain the results.

Patient information websites, patient information articles, and patient information leaflets were included as part of this study. We excluded promotional/industry articles, multimedia sources (e.g., videos), news articles, and articles aimed at healthcare professionals.

Overall, 84 websites and articles were gathered after the removal of duplicates. These were further analysed to ensure compliance with the inclusion/exclusion criteria. One video, one industry article, four websites that failed to load, six irrelevant articles, one promotional article, and 23 professional articles were excluded, which resulted in the inclusion of 48 articles for analysis (57.14%).

All articles were either in the form of a web page or a downloadable portable document format (pdf). These were analysed using a specialised website (www.readable.com), which calculated multiple scores that indicated the readability of the article. For the purposes of this study, three well-established scores were used. These included the Flesch-Kincaid Grade (FKG), which assesses the approximate reading grade level of a text, the Flesch Reading Ease (FRE), which tells you roughly what level of education someone will need to be able to read a piece of text easily, and the Gunning Fog Index (GFI), which estimates the years of formal education the reader requires to understand the text on first reading [10-12]. The formulas for these scores are listed in Table 1.

**Table 1:** Readability score formulas W/S = total words/total sentences; Sy/W = total syllables/total words; W/S = words/sentences; CW/W = complex words (three syllables or more)/words.

Name	Description	Formula
Flesch-Kincaid Grade (FKG)	A score equivalent to US grade level, e.g., FKG = 8 is equivalent to the US grade 8 standard of text	FKG = [0.39 × (W/S)] + [11.8 × (Sy/W)] – 15.5
Flesch Reading Ease (FRE)	Scored between 0 and 100. A higher score implies an easier-to-read text. Scores of 70-80 are roughly equivalent to US grade 7	FRE = 206.835 – [84.6 × (Sy/W)] – [1.015 × (W/S)]
Gunning Fog Index (GFI)	Scored 0-20 and estimates the level of education required to understand the text. A score of 6 is readable by 6th graders whereas a score of 17 is graduate level	GFI = 0.4 × [(W/S) + (CW/W) × 100]

In addition to the readability scores, understandability was evaluated using the Patient Education Materials Assessment Tool (PEMAT). This is a simple tool that was developed in recognition of the shortcomings of readability formulas to assess understandability and actionability. The tool consists of 26 items spread across two scales: understandability (19 items) and actionability (seven items). It also comes in two formats PEMAT-P for printable materials and PEMAT-A/V for audio-visual materials [13]. For the purposes of this study, we utilised PEMAT-P and considered only the understandability scale. The user guide found on the Agency for Healthcare Research and Quality website was followed for calculating the PEMAT scores for all included articles.

All 48 PEMAT scores for the evaluated articles can be provided by the author upon request.

## Results

Reading level

The mean FKG was 7.92 ± 1.91, the mean FRE was 57.92 ± 12.26, and the mean GFI was 9.35 ± 2.36. The FKG scores showed that while 16 articles were at or below the recommended reading level of 6th grade, the remaining 32 articles (67%) were at a reading level higher than that recommended by health literacy experts. On a positive note, only one article was deemed to be at a reading level higher than 10th grade with none of the articles falling into college-level reading grades. The full results are listed in Table 2.

**Table 2:** Flesch-Kincaid Grade scoring breakdown

Flesch-Kincaid Grade	US school level	Number of articles
5	5^th^ grade	9
6	6^th^ grade	7
7	7^th^ grade	12
8	8^th^ grade	4
9	9^th^ grade	8
10	10^th^ grade	7
11	11^th^ grade	0
12	12^th^ grade	1
13	College freshman	0
14	College sophomore	0

The FRE found that only one article was grade 6 level while all others were classed as more difficult to read. A significant number of articles (10 articles = 21%) were found to have readability scores that indicated a college student's level of reading ability. The full results can be seen in Table 3.

**Table 3:** Flesch Reading Ease scoring breakdown

Flesch Reading Ease	US school level	Number of articles
<10	Professional	0
10-20	College graduate	0
20-30	College graduate	1
30-40	College student	3
40-50	College student	7
50-60	10-12^th^ grade	17
60-70	8^th^/9^th^ grade	12
70-80	7^th^ grade	7
80-90	6^th^ grade	1
90-100	5^th^ grade	0

The GFI scores showed that only six articles were at a reading level of US grade 6 or below. The majority of the articles had a reading level equivalent to US grade 7-10, which is much higher than that recommended by experts for health information articles directed to the general public. The full breakdown of GFI scores is displayed in Table 4.

**Table 4:** Gunning Fog Index scoring breakdown

Gunning Fog Index	US grade level	Number of articles
0-5	5^th^ grade or below	5
6	6^th^ grade	1
7	7^th^ grade	6
8	8^th^ grade	9
9	9^th^ grade	8
10	10^th^ grade	9
11	11^th^ grade	3
12	12^th^ grade	4
13-15	College student	3
16	College graduate	0
17+	Post-graduate	0

Understandability

Understandability was assessed using the PEMAT-P scoring tool. All 48 articles were individually assessed and scored as per the user guide. While readability assesses the ease of reading a body of text, understandability focuses on comprehension of that text and the ability to process key messages. The developers of PEMAT-P have suggested a score of <70% as indicating poor understandability, whereas a score of >70% indicates good understandability.

Mean understandability was 69.63% ± 11.55%. A total of 26 articles had a score <70%, leaving 22 articles with a score above 70%.

A combination of the results to evaluate the number of web pages/articles with a desirable readability score (FRE, GFI, or FKG) and an understandability score >70% revealed only 10 met both criteria. Of these, none met both FRE and understandability criteria, two met GFI, FKG, and understandability, while eight met both FKG and understandability alone.

## Discussion

The global utilisation of digital tools and media to disseminate health messages, provide health-related information, and facilitate access to healthcare services is increasing. Individuals often seek search engines such as Google, Yahoo, and Bing to source health-related information. This increase in information-seeking behaviour has placed increased demands on health literacy for both caregivers and patients [14]. The Centers for Disease Control and Prevention have defined personal health literacy as “the degree to which individuals have the ability to find, understand, and use information and services to inform health-related decisions and actions for themselves and others”.

Low health literacy has been consistently associated with poorer ability to interpret health messages, higher mortality rates, and poorer overall health. Those of a lower socioeconomic status and with an education lower than high school have been widely documented as having poorer health literacy [15,16].

According to the Central Statistics Office in Ireland, in 2019, 91% of households had internet access; however, if the trend is followed, this figure is likely higher today in 2023. As a result, ensuring high-quality information regarding healthcare topics is available online is paramount. To allow health-related information to be accessible to as wide an audience as possible, experts have recommended that simple language and a reading level equivalent to US grade 6 or below be used [9].

Our study evaluated health information related to paediatric in-toeing as this is a common referral to orthopaedic surgeons and is often associated with significant parental concern [17]. We chose to employ the three most frequently utilised search engines to retrieve web pages and articles for our study. This approach enabled us to examine the information accessible to the majority of patients seeking to acquire knowledge on this topic. We also used 50 of the top results from each of the three search engines to limit web pages/articles being missed.

Unsurprisingly, there was an overlap between the results from the three search engines; however, 84 unique web pages/articles were still obtained. Our assessment found that over 40% of web pages/articles (36 out of 84) were not relevant to the search with the majority of irrelevant articles being those aimed at healthcare professionals rather than patients/carers. Of those that were aimed at patients, only 21% were at a desirable readability and understandability level. However, excluding duplicates, if we include all the web pages/articles (84) that were obtained, we find that only 12% of articles were at the desired readability and understandability level required for health information articles.

Our study highlights the lack of quality in healthcare literature available online to patients and emphasises the publication of improved patient information web pages. However, there are a number of limitations of this study that should be considered when evaluating the results.

Firstly, all three online searches were carried out in one region by one author. Given that search results are based on previous browsing history and location, it is possible that some relevant web pages/articles were missed or indeed favoured more heavily due to previous visits to those websites.

Secondly, the readability formulae are limited, in that they may produce a score resembling a more complex article despite simple language being used. For example, a complex word in the GFI is one which has three or more syllables. This would imply the words “interesting” and “surprising” are complex; however, most individuals would not consider this to be the case. Conversely, medical acronyms that may indeed be interpreted as complex by individuals are deemed as not complex since they are not more than three syllables.

Finally, the search was carried out exclusively in the English language. Therefore, the quality of material available or the lack thereof in other languages cannot be commented on from our results.

However, there are strengths to this study that are also to be considered. Firstly, in contrast to previous studies, both readability and understandability were assessed by the authors to give a more complete picture of the quality of healthcare materials available to patients regarding in-toeing. This is the first study of this nature to do so. This meant we were able to assess not only whether the information was easily readable but also whether the information given was understandable. Secondly, to counter the variation in online search results due to location and user, a higher number of results than previous studies were included. By including 50 results from each source, we minimised the potential for the exclusion of certain web pages/articles.

## Conclusions

Our research has brought to light significant shortcomings in online patient education resources pertaining to paediatric in-toeing. In a majority of cases, the readability of the materials surpasses the comprehension level of the average patient, making them inaccessible and unhelpful to a wide audience. Moreover, our study has revealed that the overall understandability of these online resources is lower than accepted standards. This implies that not only are most resources difficult to read but are also difficult to understand.

In light of our understanding of health literacy levels and their influence on patient outcomes, it is imperative that we expeditiously address these shortcomings. With an increasing number of patients relying on the internet for health information, it becomes crucial to significantly enhance both the readability and overall understandability of online resources related to paediatric in-toeing.

## References

[1] Burrus MT, Werner BC, Starman JS, Kurkis GM, Pierre JM, Diduch DR, Hart JM (2017). Patient perceptions and current trends in internet use by orthopedic outpatients. HSS J.

[2] Kıvrak A, Ulusoy İ (2023). How high is the quality of the videos about children's elbow fractures on YouTube?. J Orthop Surg Res.

[3] Parsa A, Prabhavalkar ON, Saeed S, Nerys-Figueroa J, Carbone A, Domb BG (2024). Best practices on patient education materials in hip surgery based on learnings from major hip centers and societies. [PREPRINT]. J Hip Preserv Surg.

[4] Hansberry DR, Agarwal N, Shah R (2014). Analysis of the readability of patient education materials from surgical subspecialties. Laryngoscope.

[5] Institute of Medicine (2004). Health Literacy: A Prescription to End Confusion. Health Literacy: A Prescription to End Confusion.

[6] Liu C, Wang D, Liu C (2020). What is the meaning of health literacy? A systematic review and qualitative synthesis. Fam Med Community Health.

[7] Murphy B, Irwin S, Condon F, Kennedy C (2022). Readability and quality of online information for patients pertaining to revision knee arthroplasty: an objective analysis. Surgeon.

[8] Oliffe M, Thompson E, Johnston J, Freeman D, Bagga H, Wong PK (2019). Assessing the readability and patient comprehension of rheumatology medicine information sheets: a cross-sectional health literacy study. BMJ Open.

[9] Cotugna N, Vickery CE, Carpenter-Haefele KM (2005). Evaluation of literacy level of patient education pages in health-related journals. J Community Health.

[10] Świeczkowski D, Kułacz S (2021). The use of the Gunning Fog Index to evaluate the readability of Polish and English drug leaflets in the context of health literacy challenges in medical linguistics: an exploratory study. Cardiol J.

[11] Kher A, Johnson S, Griffith R (2017). Readability assessment of online patient education material on congestive heart failure. Adv Prev Med.

[12] Shoemaker SJ, Wolf MS, Brach C (2014). Development of the Patient Education Materials Assessment Tool (PEMAT): a new measure of understandability and actionability for print and audiovisual patient information. Patient Educ Couns.

[13] Ulusoy I, Yılmaz M, Kıvrak A (2023). How efficient is ChatGPT in accessing accurate and quality health-related information?. Cureus.

[14] Sundell E, Wångdahl J, Grauman Å (2022). Health literacy and digital health information-seeking behavior - a cross-sectional study among highly educated Swedes. BMC Public Health.

[15] Berkman ND, Sheridan SL, Donahue KE, Halpern DJ, Crotty K (2011). Low health literacy and health outcomes: an updated systematic review. Ann Intern Med.

[16] Hickey KT, Masterson Creber RM, Reading M, Sciacca RR, Riga TC, Frulla AP, Casida JM (2018). Low health literacy: implications for managing cardiac patients in practice. Nurse Pract.

[17] Harris E (2013). The intoeing child: etiology, prognosis, and current treatment options. Clin Podiatr Med Surg.

